# Temporary extracorporeal life support: single-centre experience with a new concept

**DOI:** 10.1093/icvts/ivae043

**Published:** 2024-03-15

**Authors:** Gaik Nersesian, Daniel Lewin, Sascha Ott, Felix Schoenrath, Yuriy Hrytsyna, Christoph Starck, Frank Spillmann, Benjamin O'Brien, Volkmar Falk, Evgenij Potapov, Pia Lanmueller

**Affiliations:** Department of Cardiothoracic and Vascular Surgery, Deutsches Herzzentrum der Charité (DHZC), Berlin, Germany; DZHK (German Centre for Cardiovascular Research), Partner Site Berlin, Berlin, Germany; Department of Cardiothoracic and Vascular Surgery, Deutsches Herzzentrum der Charité (DHZC), Berlin, Germany; DZHK (German Centre for Cardiovascular Research), Partner Site Berlin, Berlin, Germany; Department of Cardiac Anesthesiology and Intensive Care Medicine, Deutsches Herzzentrum der Charité (DHZC), Berlin, Germany; Department of Cardiothoracic and Vascular Surgery, Deutsches Herzzentrum der Charité (DHZC), Berlin, Germany; DZHK (German Centre for Cardiovascular Research), Partner Site Berlin, Berlin, Germany; Department of Cardiothoracic and Vascular Surgery, Deutsches Herzzentrum der Charité (DHZC), Berlin, Germany; Department of Cardiothoracic and Vascular Surgery, Deutsches Herzzentrum der Charité (DHZC), Berlin, Germany; DZHK (German Centre for Cardiovascular Research), Partner Site Berlin, Berlin, Germany; Department of Internal Medicine and Cardiology, Charité—Universitätsmedizin Berlin, Berlin, Germany; DZHK (German Centre for Cardiovascular Research), Partner Site Berlin, Berlin, Germany; Department of Cardiac Anesthesiology and Intensive Care Medicine, Deutsches Herzzentrum der Charité (DHZC), Berlin, Germany; William Harvey Research Institute, London, UK; Berlin Institute of Health at Charité - Universitätsmedizin Berlin, Berlin, Germany; Department of Cardiothoracic and Vascular Surgery, Deutsches Herzzentrum der Charité (DHZC), Berlin, Germany; DZHK (German Centre for Cardiovascular Research), Partner Site Berlin, Berlin, Germany; Berlin Institute of Health at Charité - Universitätsmedizin Berlin, Berlin, Germany; Department of Health Sciences and Technology, Institute of Translational Medicine, Translational Cardiovascular Technologies, Swiss Federal Institute of Technology (ETH) Zurich, Zurich, Switzerland; Department of Cardiothoracic and Vascular Surgery, Deutsches Herzzentrum der Charité (DHZC), Berlin, Germany; DZHK (German Centre for Cardiovascular Research), Partner Site Berlin, Berlin, Germany; Department of Cardiothoracic and Vascular Surgery, Deutsches Herzzentrum der Charité (DHZC), Berlin, Germany; DZHK (German Centre for Cardiovascular Research), Partner Site Berlin, Berlin, Germany

**Keywords:** Impella, Extracorporeal life support, Veno-arterial extracorporeal membrane oxygenation, ECMELLA, Cardiogenic shock

## Abstract

**OBJECTIVES:**

The combination of veno-arterial extracorporeal membrane oxygenation with a micro-axial flow pump (ECMELLA) is increasingly used for cardiogenic shock (CS) therapy. We report our experience with a novel single-artery access ECMELLA setup with either femoral (2.0) or jugular venous cannulation (2.1), respectively.

**METHODS:**

Data from 67 consecutive CS patients treated with ECMELLA 2.0 (*n* = 56) and 2.1 (*n* = 11) from December 2020 and December 2022 in a tertiary cardiac center were retrospectively analyzed.

**RESULTS:**

The mean age was 60.7 ± 11 years, 56 patients (84%) were male. CS aetiology was acute on chronic heart failure (*n* = 35, 52%), myocardial infarction (*n* = 13, 19.5%), postcardiotomy syndrome (*n* = 16, 24%) and myocarditis (*n* = 3, 4.5%). Preoperatively 31 patients (46%) were resuscitated, 53 (79%) were on a ventilator and 60 (90%) were on inotropic support. The median vasoactive inotropic score was 32, and the mean arterial lactate was 8.1 mmol/l. In 39 patients (58%), veno-arterial extracorporeal membrane oxygenation was explanted after a median ECMELLA support of 4 days. Myocardial recovery was achieved in 18 patients (27%), transition to a durable left ventricular assist device in 16 (24%). Thirty-three patients (*n* = 33; 49%) died on support (25 on ECMELLA and 8 on Impella after de-escalation), 9 (13%) of whom were palliated. Axillary access site bleeding occurred in 9 patients (13.5%), upper limb ischaemia requiring surgical revision in 3 (4.5%). Axillary site infection occurred in 6 cases (9%), and perioperative stroke in 10 (15%; 6 hemorrhagic, 4 thromboembolic).

**CONCLUSIONS:**

ECMELLA 2.0/2.1 is a feasible and effective therapy for severe CS. The single-artery cannulation technique is associated with a relatively low rate of access-related complications.

**Figure:**
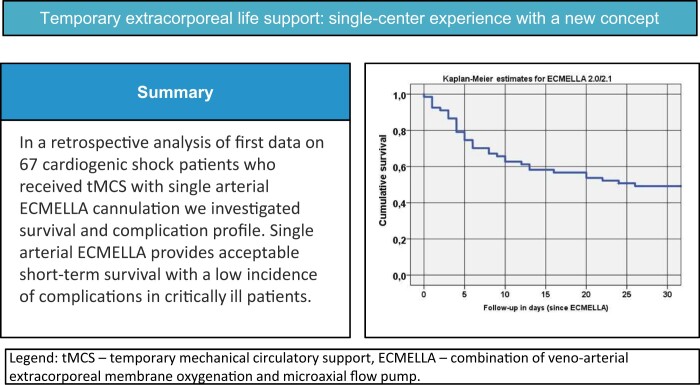

## INTRODUCTION

Mechanical circulatory support (MCS) has been used for the treatment of cardiogenic shock (CS) since the early 1960s [1]. In recent years, technological advances have revolutionized the field of extracorporeal life support (ECLS), offering more reliable and miniature devices that can provide prolonged haemodynamic support and may improve patient outcomes [1]. The benefits of MCS in improving haemodynamics in CS therapy were partly outweighed by serious device- and procedure-related complications [2, 3]. As a result of historic studies with neutral outcomes, current heart failure guidelines are restrictive with recommendations for MCS in CS [1, 4].

Currently, one of the most commonly used ECLS devices is veno-arterial extracorporeal membrane oxygenation (va-ECMO) [4]. This readily available system can be implanted even during ongoing cardiopulmonary resuscitation (CPR) and has significantly improved acute survival for CS patients [5]. Prolonged va-ECMO support is associated with an increased risk of complications, in particular vascular complications and bleeding [6, 7]. Another important drawback is the increased left ventricular afterload due to retrograde flow during extracorporeal membrane oxygenation (ECMO) support, which can result in pulmonary venous congestion and lung oedema [8]. To achieve left ventricular unloading of the failing ventricle, surgical venting, intra-aortic balloon pumps or micro-axial flow pumps (mAFP) are used concomitantly with va-ECMO [8, 9].

The combination of va-ECMO and Impella (Abiomed, Denvers, MA, USA), known as the ECMELLA approach, is associated with a significant survival benefit compared to va-ECMO alone [2, 9–11]. However, studies have shown a higher incidence of bleeding complications, which is likely due to the need for an additional arterial access for Impella placement [2].

In this study, we present our experience with the novel ECMELLA 2.0/2.1 approach, in which a single arterial access technique is used for the placement of both the Impella and the arterial cannula of the ECMO system.

## PATIENTS AND METHODS

### Ethics statement

The presented study is a part of a register-based research approved by the institutional ethics committee of the Cahrité Universitaetsmedizin Berlin (EA2/196/21: version 03/2023). The requirement for informed consent was waived, as all patients in the database were anonymized. All medical records were retrospectively reviewed.

### Study population

We collected and retrospectively analyzed data of 67 consecutive CS patients treated with 2.0 (*n* = 56) and 2.1 (*n* = 11) approaches at a high-volume tertiary care center between December 2020 and December 2022.

### Data collection

Demographic data, clinical data, as well as last available haemodynamic data and laboratory values prior to ECMELLA 2.0/2.1 implantation were retrospectively collected from patients’ electronic health records and analyzed.

The vasoactive-inotropic score was calculated using the following formula [12]:

Dopamine+dobutamine+milrinone (×10)+epinephrine (×100)+norepinephrine (×100) (all μg/kg/min)+vasopressin (×10 000) (IU/kg/min)

The Model of End-Stage Liver Disease XI score was calculated using the formula [13]:

5.11×ln(bilirubin)+11.79×ln(creatinine)+9.44

The SAVE (Survival After Veno-Arterial ECMO) score was calculated by inputting previously described preoperative parameters into the official online calculator (https://www.elso.org/savescore/index.html) [14]. As the score has not been validated for postcardiotomy patients, no SAVE score was calculated for this patient group. Based on the SAVE score a median in-hospital survival probability was calculated for all other patient groups.

Complications on temporary mechanical circulatory support (tMCS) were assessed according to Extracorporeal Life Support Organization definitions[6]. Haemolysis was defined as blood-free haemoglobin of ≥60 mg/dl in 1 measurement with or without clinical signs of hemolysis (dark hemolytic urine, icterus). Prosthetic graft infections were assessed on the basis of clinical manifestation (signs of local inflammation, wound dehiscence or purulence) as well as laboratory, microbiological and radiological findings [15].

### Statistical analysis

Continuous variables were tested for a normal distribution using the Kolmogorov-Smirnov test and are presented as median (interquartile range) or mean (± standard deviation), respectively. The *t*-test for independent samples and the Mann–Whitney *U* test were used to compare continuous variables. Categorical variables are presented as *n* (%). Categorical variables were compared using the Chi-squared test.

Univariate logistic regression analysis was performed to predict risk factors for 30-day mortality. The odds ratios (OR) with their 95% confidence intervals (CIs) were calculated for relevant risk factors. Parameters with two-sided *P*-values <0.05 were considered statistically significant.

Overall survival and survival in different subgroups were analyzed using Kaplan–Meier estimates with 95% CIs. Log-rank testing was used to compare patient groups.

The primary end point was 30-day survival. Secondary clinical end points included in-hospital survival and survival on support as well as postoperative complications (e.g., bleeding, hemolysis, limb ischaemia).

Analyses were exploratory in nature; there was no prespecified plan to adjust for multiple comparisons.

All tests were performed using IBM SPSS Statistics for Windows, Version 25.0, Armonk, NY: IBM Corp.

### Surgical technique

The surgical technique has been described in detail in previous publications [16, 17]. Briefly, the axillary artery is surgically exposed through a 3- to 5-cm incision in the deltopectoral groove. After administering heparin (usually 5000–7500 IU, target ACT 200 s), the artery is partially clamped and longitudinally incised. A 10-mm Y-shaped aorto-bifemoral vascular prosthesis (GELSOFT Vascutek AX Bifem REF 6510, Terumo; Terumo Heart Inc., Ann Arbor, MI, USA) is then anastomosed in end-to-side technique with the axillary artery using a running 5–0 Prolene suture. The vascular prosthesis is subsequently pulled out caudally from the wound through a separate 1-cm incision. The Impella 5.5 (previously Impella 5.0) (Abiomed, Danvers, MA, USA) mAFP is then implanted as described [16, 17]. Finally, the arterial cannula of the va-ECMO is placed into the brachiocephalic artery through the other branch of the Y-prosthesis via a guidewire under fluoroscopic control [16, 17].

The contraindications for single arterial cannulation of the axillary artery included: small vessel size (<5 mm), presence of severe calcifications, vessel dissection, technical features precluding Impella placement [mechanical aortic valve, presence of an aortic arch stent or mobile thrombus in the left ventricle (LV)]. In patients undergoing CPR, the first step was to stabilize the patient on peripheral ECMO (extracorporeal CPR), after which the ECMELLA single arterial access was established while the arterial cannula was switched from the groin to the axillary artery. Primary ECMELLA implantation under chest massage is not recommended due to technical challenging and time-consuming circumstances [18].

**Figure 1:**
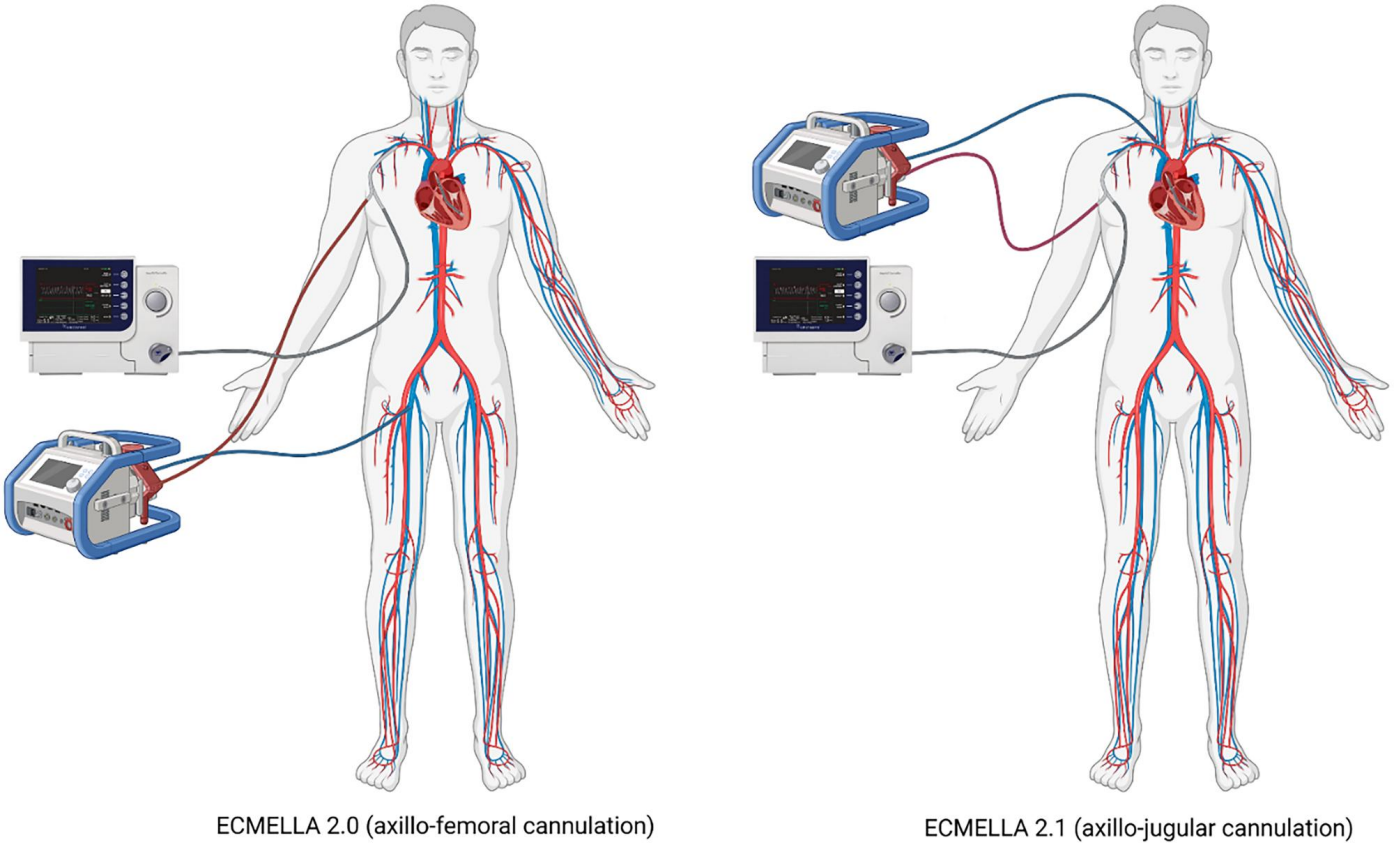
ECMELLA 2.0/2.1 cannulation technique.

Venous cannulation can be performed via the femoral (ECMELLA 2.0) or the right jugular (ECMELLA 2.1) access (Fig. 1). The decision whether to perform femoral or jugular cannulation was made individually in each case. The ECMELLA 2.1 approach is preferred in patients in whom prolonged tMCS with ECMELLA is anticipated (e.g. acute myocarditis, bridge to transplant) in order to facilitate the patient’s mobilization. The right jugular vein represents a feasible alternative access site in patients in whom femoral cannulation is not possible or is associated with an increased complication rate (s/p previous interventions via an inguinal access, infections, s/p vascular complications). Thrombus formation in the right jugular vein or the need for central line access precludes placement of a venous cannula. Jugular vein cannulation is performed either under echocardiographic or fluoroscopic guidance. If necessary, a switch from the femoral to the jugular vein during support may be performed to enhance patient mobilization.

If the patient is already supported by an mAFP inserted via the axillary artery, an upgrade to ECMELLA 2.0/2.1 can be established using the existing vascular access. In this case, a 10-mm vascular prosthesis is anastomosed end-to-side with the pre-existing one and is subsequently used for inserting the arterial cannula of the va-ECMO. Before placing the cannula, a retrograde flush and/or thrombectomy of the vascular graft should be performed to prevent thrombus migration into the circulation.

It is important to note that high blood flow via the axillary artery can lead to arm hyperperfusion. To mitigate this, the arterial cannula of the va-ECMO should be placed proximally to the anastomosis. Careful monitoring of hand perfusion using clinical parameters, invasive blood pressure measurement, near-infrared spectroscopy or oxygen saturation measurement should be established to avoid hand ischaemia.

## RESULTS

### ECMELLA modalities

**Figure 2:**
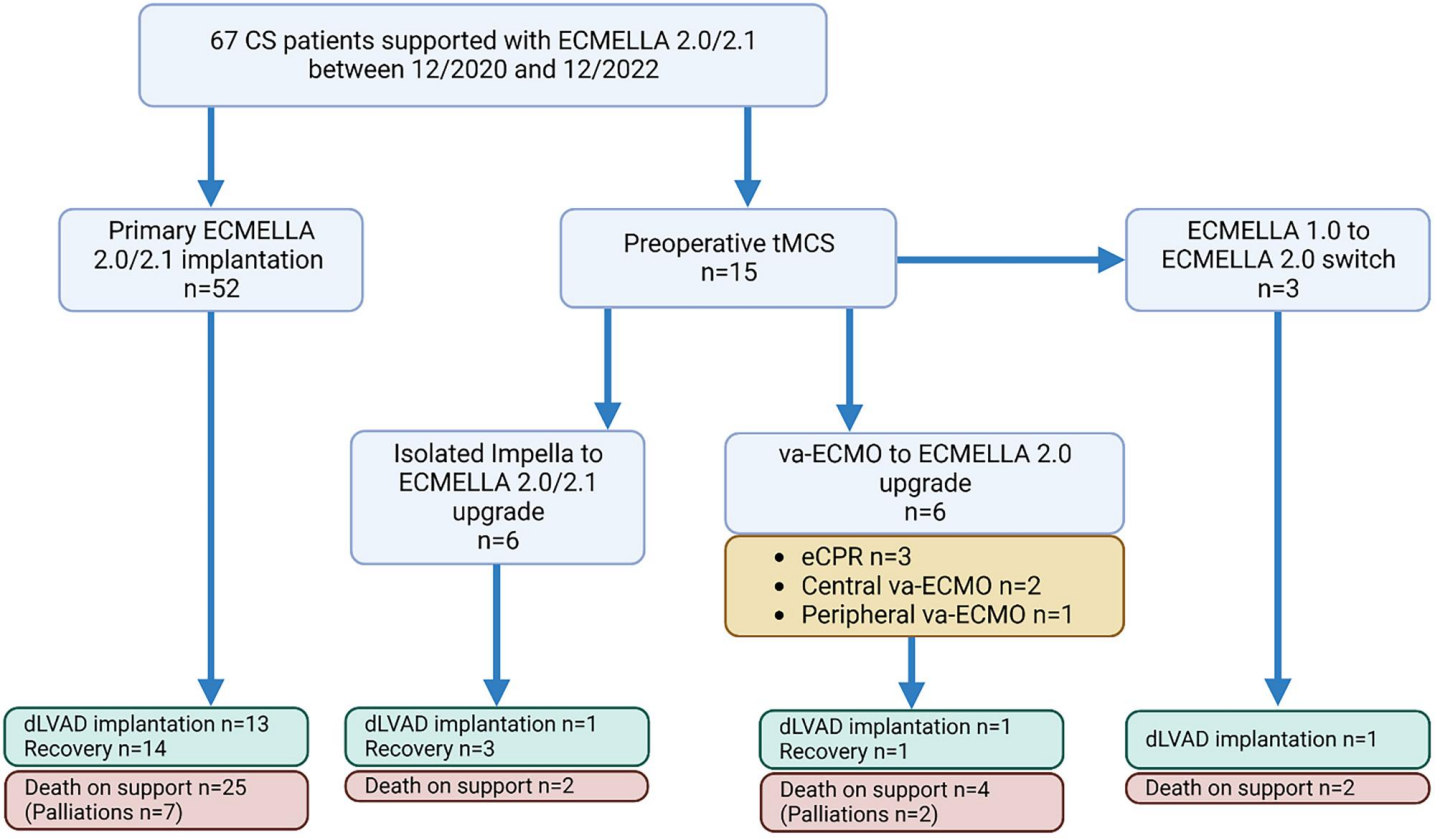
ECMELLA modalities and outcomes. dLVAD: durable left ventricular assist device; eCPR: extracorporeal cardiopulmonary resuscitation; tMCS: temporary mechanical circulatory support; va-ECMO: veno-arterial extracorporeal membrane oxygenation.

During the study period, 67 patients were treated for CS with the single arterial ECMELLA approach; in 56 cases (84%) ECMELLA 2.0 and in 11 (16%) ECMELLA 2.1 implantation was performed. Fifteen patients (22%) were on tMCS at the time of establishing ECMELLA 2.0/2.1: 6 (9%) on Impella, 6 (9%) on va-ECMO and 3 (4%) were switched from conventional ECMELLA to a single arterial access approach due to femoral complications (Fig. 2).

In 3 patients (4%), va-ECMO implantation with femoral cannulation was performed under CPR, pursuing extracorporeal CPR strategy; 2 patients presented with centrally cannulated va-ECMO.

In all patients, a surgically implanted Impella 5.0/5.5 device was used for ECMELLA. In 17 cases (25%), a percutaneous Impella model (CP or 2.5) had been previously placed but was removed and replaced by a larger mAFP during ECMELLA 2.0/2.1 implantation due to complications and/or to facilitate de-escalation later on.

Preoperative demographics and laboratory parameters are presented in Tables 1 and 2.

**Table 1:** Preoperative demographic parameters

Parameter	Value	Missing data (%)
Male sex, *n* (%)	56 (83.6%)	0
Age (years), mean ± SD	60.7 ± 11	0
BMI (kg/m^2^), mean ± SD	29.1 ± 6.4	1.5
Arterial hypertension, *n* (%)	45 (67.2%)	3
Preoperative atrial fibrillation, *n* (%)	30 (44.8%)	0
Diabetes mellitus, *n* (%)	25 (37.3%)	4.5
IDDM, *n* (%)	10 (15%)	4.5
Coronary artery disease, *n* (%)	48 (72%)	0
History of myocardial infarction, *n* (%)	36 (53.8%)	1.5
STEMI, *n* (%)	18 (26.9%)	10.5
Chronic kidney disease, *n* (%)	25 (37.3%)	4.5
Preoperative renal replacement therapy, *n* (%)	3 (4.5%)	0
COPD, *n* (%)	8 (12%)	6
Pulmonary hypertension, *n* (%)	6 (9%)	7.5
Peripheral arterial disease, *n* (%)	8 (12%)	1.5
Cerebral arterial disease, *n* (%)	2 (3%)	1.5
LVEF (%), median [IQR]	18 [10; 23]	18
LVEDD (mm), median [IQR]	58 [51; 68]	13
Preoperative invasive ventilation, *n* (%)	53 (79.1%)	0
Preoperative inotropic support, *n* (%)	60 (89.5%)	10.5
Preoperative VIS score, median [IQR]	32 [20–73]	0
Preoperative CPR, *n* (%)	31 (46.3)	0

BMI: body mass index; COPD: chronic obstructive pulmonary disease; CPR: cardiopulmonary resuscitation; IDDM: insulin-dependent diabetes mellitus; IQR: interquartile range; LVEDD: left ventricular end-diastolic diameter; LVEF: left ventricular ejection fraction; SD: standard deviation; STEMI: ST-elevation myocardial infarction; VIS: vasoactive inotropic score.

**Table 2:** Preoperative laboratory parameters

Parameter	Value	Missing data (%)
WBC (K/µl), median [IQR]	15 [10.3; 21.9]	12
Haemoglobin (g/dl), median [IQR]	11.7 [9; 14]	12
Haematocrit (%), median [IQR]	34.7 [28; 40]	12
PLC (K/µl), median [IQR]	196 [133; 237]	12
CRP (mg/dl), median [IQR]	5 [1.8; 11.3]	21
Albumin (g/dl), mean ± SD	2.9 ± 0.6	28
aPTT (s), median [IQR]	52 [43; 155]	16
INR, median [IQR]	1.7 [1.4; 2.6]	16
CK (U/l), median [IQR]	408 [145; 1597]	13
CK-MB (U/l), median [IQR]	82 [43; 254]	16
GOT (U/l), median [IQR]	389 [118; 1932]	18
LDH (U/l), median [IQR]	964 [502; 2184]	16
Lipase (U/l), median [IQR]	45.6 [24.9; 135.3]	27
Creatinine (mg/dl), median [IQR]	2.0 [1.4; 2.4]	15
Urea (mg/dl), median [IQR]	62 [41; 87]	15
Creatinine clearance (ml/min/1.73 m^2^), mean ± SD	36.9 ± 18.1	15
Total bilirubin (mg/dl), median [IQR]	1.2 [0.6; 2.6]	16
MELD-XI score, mean ± SD	20.1 ± 6.8	16
pH, mean ± SD	7.27 ± 0.11	0
pO_2_ (mmHg), median [IQR]	148 [95; 327]	0
pCO_2_ (mmHg), median [IQR]	42.5 [33; 50]	0
${\text{HCO}}_{3}^{-}$ (mmol/l), mean ± SD	20.0 ± 5.2	0
BE (mmol/l), median [IQR]	−6 [-10; -1.8]	0
Potassium (mmol/l), mean ± SD	4.5 ± 0.9	0
Sodium (mmol/l), mean ± SD	140 ± 7.2	0
Glucose (mg/dl), median [IQR]	155 [114; 210]	0
Lactate (mmol/l), median [IQR]	8.1 [2.6; 11.9]	0

aPTT: activated partial thromboplastin time; BE: base excess; CK: creatine kinase; CK-MB: myocardial specific creatine kinase isoenzyme; CRP: C-reactive protein; GOT: glutamic-oxaloacetic transaminase; INR: international normalized ratio; IQR: interquartile range; LDH: lactate dehydrogenase; MELD-XI: Model of End-Stage Liver Disease XI; PLC: platelet count; SD: standard deviation; WBC: white blood cell.

### Cardiogenic shock aetiology

Acute on chronic heart failure was the leading aetiology of CS in our cohort (*n* = 35, 52%), followed by postcardiotomy syndrome (*n* = 16, 24%), acute myocardial infarction (*n* = 13, 19.5%) and fulminant myocarditis (*n* = 3, 4.5%).

Sixteen patients (24%) had undergone cardiac surgery within 7 days prior to tMCS initialization (postcardiotomy cases), and 10 patients (15%) could not be weaned from cardiopulmonary bypass and required intraoperative tMCS. Five patients (7.5%) underwent emergency coronary artery bypass grafting alone or in combination with valve surgery, and 4 patients (6%) required urgent valve surgery due to endocarditis. The remaining 7 cases (10%) were scheduled for an elective cardiac procedure. Twelve out of 16 patients underwent primary ECMELLA 2.0 implantation: in 6 cases intraoperatively and in the other 6 cases after a median of 16 h post-surgery. The remaining 4 cases received intraoperative tMCS with Impella, va-ECMO or ECMELLA 1.0 with a subsequent upgrade to ECMELLA 2.0.

### Outcomes

Of the 67 patients, 48 (72%) recovered from the initial shock as indicated by normalization of lactate levels, cessation of vasopressors and/or de-escalation of tMCS (e.g. va-ECMO weaning and removal). In 39 patients (58%) va-ECMO was successfully explanted after a median ECMELLA support period of 4 days. Twenty-two (33%) patients could be extubated, 21 of them on ongoing tMCS, while 14 other patients (21%) required tracheostomy for prolonged ventilation; the remaining 31 patients (46%) died on mechanical ventilation.

Sustained myocardial recovery and complete weaning from tMCS (removal of both va-ECMO and mAFP) was achieved in 18 cases (27%). Durable left ventricular assist device (LVAD) implantation was performed in 16 patients (24%); 13 implantations (19.5%) were performed on Impella support and 3 (4.5%) directly on ECMELLA. All LVAD implantations were performed on cardiopulmonary bypass in order to facilitate left ventricle inspection. In this setting, tMCS was switched to cardiopulmonary bypass intraoperatively. Thirty-three patients (49%) died on support (25 on ECMELLA and 8 on Impella after de-escalation); of these, 9 (13%) received palliative care due to severe neurological damage, a lack of viable treatment options, poor chances of recovery or in line with the patients’ wishes.

**Figure 3:**
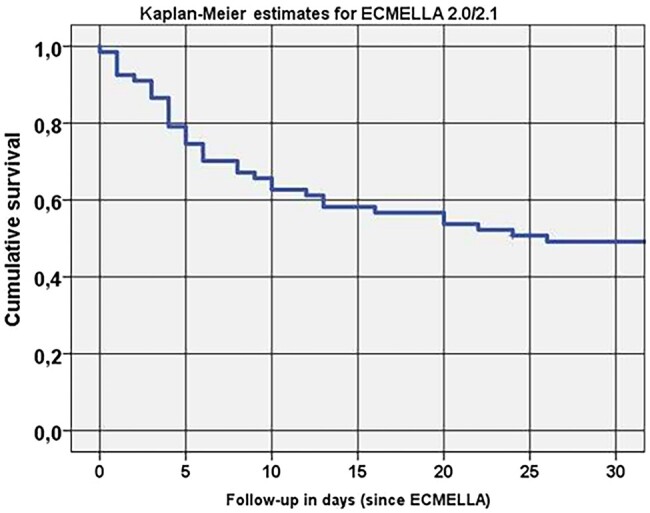
Kaplan–Meier estimates for 30-day survival.

An overall 30-day survival of 48% was achieved (Fig. 3). Among patients who underwent ECMELLA 2.0/2.1 implantation as the first-line tMCS modality, 30-day survival was 53.5%. Two patients (3%) could be discharged home directly from our clinic, 28 (48%) were transferred to a tertiary hospital, while 5 (7.5%) died after the transfer.

The 30-day survival per CS aetiology presented as follows: acute on chronic heart failure (53%), acute myocardial infarction (54%), postcardiotomy syndrome (31%) and acute myocarditis (100%).

### Complications

Surgical revision due to axillary site bleeding was necessary in 9 patients (13%). One patient experienced severe bleeding from the axillary artery, prompting recannulation to the femoral artery (Table 3).

**Table 3:** Complications during ECMELLA support

Complication	*n* (%)
Haemolysis	11 (16)
Impella dislodgement requiring explantation/exchange	4 (6)
Axillary site bleeding requiring surgical revision	9 (13)
Upper ischaemia requiring surgical revision	3 (4.5)
Respiratory tract bleeding^a^	13 (19)
GI bleeding	5 (7.5)
Stroke	10 (15)
Haemorrhagic stroke	6 (9)
Thromboembolic stroke	4 (6)
Prosthetic graft infection	6 (9)
Postoperative renal replacement therapy	26 (39)

a Including epistaxis and oropharyngeal bleeding.

GI: gastrointestinal.

On ECMELLA support 5 patients (7.5%) experienced upper limb complications (Table 3). Four suffered arm ischaemia: in 1 case, transient ischaemia recovered spontaneously and in 3 cases surgical revision was necessary. In particular, 2 patients required an arm perfusion line, and in 1 patient slight retraction of the arterial cannula of the va-ECMO was necessary. In 1 case, arm hyperperfusion prompted distal banding of the axillary artery. No complications associated with the jugular access were observed.

Prosthetic graft infections were observed in 6 patients (9%) and required surgical revision of the axillary artery with removal of the remnants of the graft prosthesis and reconstruction of the vessel. In 5 cases (7.5%), an infection was diagnosed following complete tMCS explantation after a median of 44 days (range 21–187) after ECMELLA initiation. In 1 case, an early-onset infection occurred on ECMELLA support 17 days after implantation.

Perioperative stroke was observed in 10 patients (15%), including thromboembolic stroke in 4 and hemorrhagic stroke in 6 patients, 5 of whom were preoperatively resuscitated (Table 3). In 1 case, hypoxic brain damage as a sequela of CPR was diagnosed after ECMELLA implantation.

Femoral site complications are presented in Table 4. Femoral site bleeding was observed in 9 cases (13%): 5 patients had femoral arterial cannulation (4 prior to ECMELLA 2.0/2.1, 1 after recannulation from the axillary artery due to severe bleeding). In the remaining 4 cases, femoral site bleeding was caused by a previously implanted Impella CP and by the venous cannula in 2 patients each.

**Table 4:** Complications related to femoral artery cannulation

Complication	*n* (%)
Femoral site bleeding	9 (13)
Femoral bleeding requiring surgical revision	5 (7)
Lower limb ischaemia	4 (6)
Lower limb ischaemia requiring surgical revision	3(4.5)
Lower limb compartment	4 (6)
Fasciotomy	4 (6)
Lower limb amputation	1 (1.5)
Femoral site infection	4 (6)

### Left ventricle unloading

**Figure 4:**
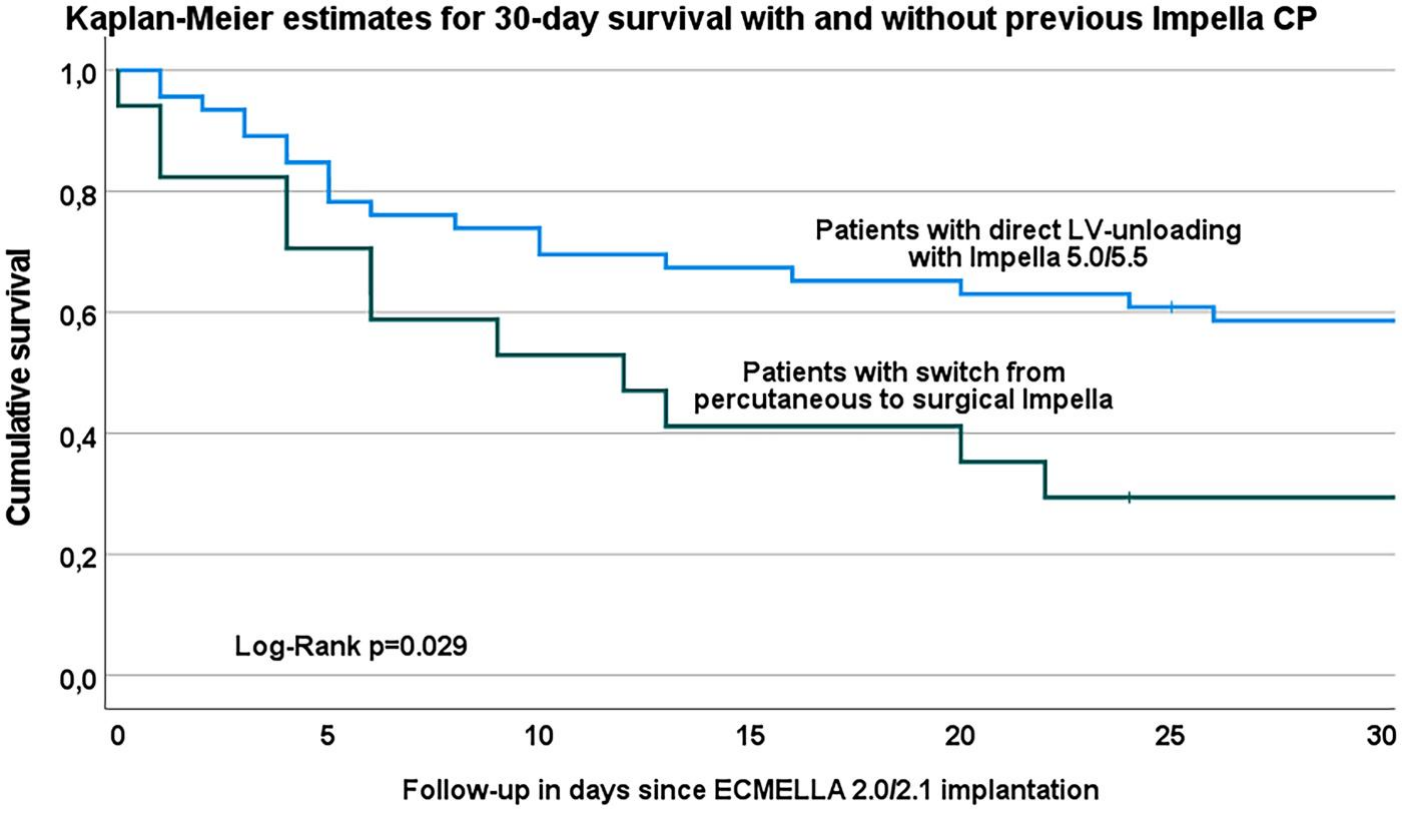
Kaplan–Meier estimates for primary Impella 5.0/5.5 or Impella CP unloading. LV: left ventricular.

After ECMELLA 2.0/2.1 insertion, a median va-ECMO and Impella 5.0/5.5 flow of 5 [5; 5] and 2 [2; 2.5] l/min was achieved, respectively, resulting in a combined support of 7 [7; 8] l/min. Sixty-three (94%) patients received first-line LV unloading (simultaneously with or prior to va-ECMO implantation). In 46 cases (69%), Impella 5.0/5.5 was primarily implanted, and in 17 (25%), previously implanted Impella CP or 2.5 was upgraded to Impella 5.0/5.5 during ECMELLA 2.0/2.1 implantation. In the remaining 4 patients, Impella 5.0/5.5 was implanted after a mean of 21 h. Patients who underwent direct LV unloading with Impella 5.0/5.5 had a significantly higher 30-day survival compared with those who were switched from percutaneous support (Impella 2.5 or CP) to Impella 5.0/5.5 (58.7% vs 29.4%, *P* = 0.029, Fig. 4).

### Risk factors

Postcardiotomy CS was associated with a significantly lower 30-day survival compared with other etiologies (31% vs 55%, *P* = 0.046); however, the relative risk was not significant (OR 2.7; 95% CI [0.8; 8.8], *P* = 0.1).

Thirty-one patients (46%) underwent CPR preoperatively during the index hospitalization. 30-Day survival between resuscitated and non-resuscitated patients was similar (51.6% vs 47.2%, *P* = 0.8, respectively, OR 0.84; 95% CI [0.3; 2.2], *P* = 0.72).

Among the patients who received direct support with ECMELLA 2.0/2.1 and had no other tMCS devices prior to implantation (*n* = 41, 62%), elevated preoperative arterial lactate and vasoactive inotropic score were not associated with an increased 30-day mortality (OR 1.17; 95% CI [0.6; 2.3], *P* = 0.66 and OR 0.62; 95% CI [0.3; 1.4], *P* = 0.3, respectively). However, all patients with lactate >16.7 mmol/l (150 mg/dl) prior to ECMELLA died on support.

## DISCUSSION

The novel single arterial access ECMELLA 2.0/2.1 approach offers optimal haemodynamic support while reducing the risk of complications associated with the need for a second arterial access site.

Our experience demonstrates that single arterial access ECMELLA is feasible in a wide range of patients suffering from severe CS [3, 19]. Recent studies have reported a 30-day survival of 39–44% in comparable populations, whereas in our non-postcardiotomy group, the survival rate was as high as 55%, even though the cohort was too small to perform a robust mortality analysis [2, 11]. The in-hospital survival in our non-postcardiotomy patients was higher than expected as calculated using the SAVE score (45% vs 30%) [14]. One possible explanation for these results could be the immediate LV unloading with a combined perfusion rate of up to 12 l/min and lower complication rates when compared with data from previous studies [9, 11, 19, 20]. This can be attributed to the single arterial access and a shorter va-ECMO duration. Still, for a clear demonstration of the benefits of the single arterial ECMELLA concept, a control group with a conventional approach is required.

In our cohort, 18 patients were successfully weaned from tMCS support and 16 were bridged to a durable LVAD. Notably, our study population consisted of critically ill patients: 46% had undergone CPR prior to MCS, the median vasoactive inotropic score in our cohort was 32, and the median arterial lactate level was 8.1 mmol/l. The 94% 30-day survival after durable LVAD implantation suggests that patients can be adequately preconditioned on ECMELLA.

In 9 cases (13%), end-of-life care was initiated due to severe brain damage or per the patients’ wishes. These included patients who could initially be stabilized on tMCS but were not eligible for durable LVAD implantation.

LVAD implantation in Interagency Registry of Mechanically Assisted Circulatory Support 1/2 patients is known to have a high postoperative mortality rate. While single-centre studies have reported 30-day survival rates ranging from 60% to 85%, there are currently no robust data available on the survival benefits of bridging patients on va-ECMO to a durable LVAD [21, 22]. Use of va-ECMO helps stabilize haemodynamics and end-organ perfusion during the acute phase of CS. However, the patients’ evaluation for LVAD candidacy should be kept short due to the risk of ECMO-related complications and limited mobilization on support [7, 21].

### Left ventricle unloading

Sufficient LV unloading has become a crucial component of contemporary va-ECMO therapy and can be achieved by employing passive and active methods [8]. However, each technique has its own benefits and complication profiles. To date, the evidence base comparing different LV unloading strategies is limited and no modality or strategy can be considered a clear gold standard [2]. Various studies have emphasized the importance of timely LV unloading in patients on va-ECMO, leading to an increased use of the ECMELLA 1.0 approach in recent years [2, 10, 11]. However, ECMELLA 1.0 therapy results in more complications compared with va-ECMO alone [2].

In our institution, the preference is to use surgically implanted Impella 5.0/5.5 devices, which provide sufficient haemodynamic support. A previous retrospective analysis conducted at our institution found no difference in outcomes between patients treated with isolated Impella CP or Impella 5.0/5.5 systems; however, when it comes to weaning from va-ECMO percutaneous mAFP systems (Impella CP)—providing only partial circulatory support with a maximum of 3.5 l/min of cardiac output–may not be the optimal choice [23]. Patients supported with percutaneous mAFP experience a higher rate of hemolysis, which could also increase the incidence of acute kidney injury with the need for renal replacement therapy [8, 11, 24]. This could be attributed to the higher shear stress of percutaneous mAFP devices, which have a smaller diameter and higher rotation speeds than larger models and difficulties in repositioning due to the large distance from the femoral artery to the LV. In our cohort, patients who received direct LV unloading with Impella 5.0/5.5 showed a significantly higher 30-day survival rate compared with those who were upgraded from percutaneous Impella 2.5 or CP devices.

In addition, early mobilization of patients (which has been shown to be beneficial in the intensive care unit setting) is not possible safely with transfemorally placed mAFPs [23].

It is important to interpret the presented results with caution, as the majority of these patients were transferred to our institution from external hospitals due to therapy-related complications or the lack of further treatment options. As a result, these patients may exhibit a selection bias and longer durations of shock. Careful consideration is required in this context when selecting the most appropriate LV unloading technique for ECMELLA patients [25].

The recently published ECLS-SHOCK trial failed to demonstrate a survival benefit in patients suffering from shock due to acute myocardial infarction undergoing myocardial revascularization and supported by percutaneous ECMO [26]. Last, but not least, the lack of LV unloading in the intervention cohort might have contributed to the absence of a survival benefit [26].

The advantages of concomitant LV unloading in va-ECMO patients have been demonstrated in various previous publications as having a significant impact on patient outcomes [8, 9, 27]. The study by Schrage *et al.* [2] demonstrated that conventional ECMELLA 1.0 patients have a better survival despite significantly higher complication rates, especially for bleeding and hemolysis, compared to va-ECMO therapy alone. Furthermore, it has been shown that delayed initiation of LV unloading >2 h after establishing va-ECMO eliminates the statistically significant survival benefit of a first-line ECMELLA strategy [2, 8, 27]. This finding was confirmed by Radakovic *et al.* [9] who compared contemporaneous, prophylactic LV unloading with an ECMELLA strategy versus a reactive bail-out approach in an already distended ventricle. They demonstrated that patients who received initial LV unloading via Impella during va-ECMO therapy showed a significantly better survival and a higher rate of myocardial recovery [9]. We therefore postulate that the ECMELLA 2.0/2.1 approach, with simultaneous initiation of ECMO and Impella treatment, provides a beneficial combination of immediate and effective LV unloading and best possible organ perfusion [17, 18, 20, 28].

### Complications

Complications associated with femoral ECMELLA (ECMELLA 1.0) therapy remain a significant concern. According to the multicentre data from Schrage *et al.* [8], percutaneous catheter pump implantation, such as Impella CP, was used in the majority (91.1%) of patients due to the ease of implantation and the availability of equipment in catheterization labs. However, this technique has been associated with a higher risk of vascular complications and access site-related bleeding [8, 11, 29]. In particular, access site-related ischaemia requiring intervention was observed by Schrage *et al.* [2] in 21.7% of ECMELLA cases, compared with 4.5% in our cohort. Severe bleeding, determined by the need for an additional vascular access for Impella placement, is twice as high in ECMELLA 1.0 patients (38.4%) than in isolated va-ECLS cohorts (18%) [2]. In our cohort, axillary site bleeding requiring surgical revision occurred in 13% of cases.

Another drawback of LV unloading with percutaneous Impella CP is the increased risk of severe hemolysis and subsequently renal injury [29]. In the study by Thevathasan *et al.* [29], patients who received LV unloading with a percutaneous Impella device presented a higher risk for hemolysis, resulting in a significant increase in the incidence of renal replacement therapy (49.1% of ECMELLA patients and 39.5% of va-ECMO patients). Similar incidences were reported by Schrage *et al.* [2] In our ECMELLA 2.0 patients, the incidence of hemolysis and renal replacement therapy were comparable with the isolated use of va-ECMO.

Axillary site infections related to the graft prosthesis were observed in ∼10% of patients supported with ECMELLA 2.0/2.1, and the vast majority of these cases occurred after tMCS removal. These findings are comparable with those among patients supported with a surgically implanted Impella alone but are remarkably lower compared with femoral va-ECMO cannulation, which has an infection rate of up to 30% [8, 15]. A notable advantage of surgical cannulation via a vascular graft is the ease of explantation at the bedside under local anaesthesia, eliminating the potential risks associated with surgical decannulation, as is required in the case of femoral va-ECMO cannulation [15]. This approach also contributes to a reduced resource consumption [15]. Since most patients did not exhibit signs of a prosthetic graft infection at the time of explantation, there is no justification for complete removal of the vascular graft and reconstruction of the axillary artery, especially considering the associated risk of brachial plexus injury [15].

The ECMELLA 2.0/2.1 technique offers the benefits of biventricular unloading, blood oxygenation and decarboxylation, as well as high-flow circulatory support, while avoiding the typical va-ECMO complications associated with femoral cannulation, such as leg ischaemia, groin bleeding and infections. In our patient population, all groin site complications, including bleeding, fasciotomy and amputation, were associated with previous femoral artery cannulation for percutaneous Impella or/and femoral va-ECMO.

### Risk factors

CPR is widely recognized as a significant risk factor for cardiac patients and has been associated with an inferior survival in patients supported solely by Impella or va-ECMO [5, 18, 25]. Published data reveal that survival rates for patients who experienced in-hospital cardiac arrest and were supported by va-ECMO range from 23% to 42% [5]. In contrast, survival rates for CPR patients supported by Impella alone remain below 20% [25, 30]. However, in our current analysis, resuscitated patients who received ECMELLA support not only demonstrated a remarkable 30-day survival rate exceeding 50% but also exhibited outcomes similar to those of the non-CPR subgroup. These findings suggest that the ECMELLA 2.0/2.1 approach may be an effective therapy for this particular patient population.

In our cohort, we observed that none of the patients with a preoperative arterial lactate level exceeding 16.7 mmol/l (150 mg/dl) survived. In our previous analysis, a lactate level above 11 mmol/l (100 mg/dl) was a critical threshold above which no patient with isolated Impella support survived [18]. Therefore, while the ECMELLA approach may increase the threshold for the point of no return, it is crucial to initiate tMCS before irreversible organ ischaemia has occurred.

### Limitations

Limitations include the retrospective nature of the study and the relatively small number of patients.

During the study period a total of 114 patients was supported with the ECMELLA approach; single-arterial cannulation was performed in 67 patients (59%). Patient assignment may have been biased due to the fact that in some patients the decision to perform primary ECMELLA 2.0/2.1 implantation or upgrade to single arterial cannulation was based on the surgeon’s preference or due to therapy-associated complications. Since its introduction in December 2020, the single-arterial cannulation method has become a well-established approach in our institution and an increasing number of surgeons have begun to implement this method. Currently, single-arterial ECMELLA is the preferable approach for cannulation in our institution, provided no contraindications are present.

## CONCLUSION

The ECMELLA 2.0/2.1 approach offers a comprehensive solution by combining the advantages of LV unloading, adequate organ perfusion and the convenience of single arterial cannulation with consequently less approach-related complications. In a cohort of 67 patients with severe CS, this strategy yielded favourable survival rates and low complication rates. To further explore the benefits and limitations of the novel single-access ECMELLA technique, future studies comparing it with conventional approaches should be conducted. These investigations will contribute to a better understanding of the potential advantages of ECMELLA and its role in the management of severe CS.

## Data Availability

The data analyzed in the study cannot be provided to a third party due to the data protection policy of our institution.

## ABBREVIATIONS

CIs: Confidence intervals
CPR: Cardiopulmonary resuscitation
CS: Cardiogenic shock
ECLS: Extracorporeal life support
LV: Left ventricle
LVAD: Durable left ventricular assist device
mAFP: Micro-axial flow pump
MCS: Mechanical circulatory support
OR: Odds ratio
SAVE score: Survival after veno-arterial ECMO score
tMCS: Temporary mechanical circulatory support
va-ECMO: Veno-arterial extracorporeal membrane oxygenation
VIS: Vasoactive inotropic score
